# Prevalence, Antimicrobial Susceptibility Pattern, and Associated Factors of Urinary Tract Infections among Pregnant and Nonpregnant Women at Public Health Facilities, Harar, Eastern Ethiopia: A Comparative Cross-Sectional Study

**DOI:** 10.1155/2020/9356865

**Published:** 2020-08-07

**Authors:** Degu Abate, Dadi Marami, Shiferaw Letta

**Affiliations:** ^1^Department of Medical Laboratory Sciences, College of Health and Medical Sciences, Haramaya University, P.O. B: 235, Harar, Ethiopia; ^2^School of Nursing and Midwifery, College of Health and Medical Sciences, Haramaya University, P.O. B: 235, Harar, Ethiopia

## Abstract

**Background:**

Urinary tract infection is one of the most common health problems worldwide, afflicting many women in reproductive age, especially in developing countries. Increased risk of infection has been attributed to pregnancy and antimicrobial resistance.

**Objective:**

To compare the prevalence, antimicrobial susceptibility pattern of the bacteria and associated factors of urinary tract infections among pregnant and nonpregnant women attending public health facilities, Harar, Eastern Ethiopia.

**Methods:**

A health facility-based comparative cross-sectional study was conducted among 651 randomly selected women from public health facilities, Harar, Eastern Ethiopia, between February 2017 and December 2017. Pertinent data were collected through a face-to-face interview using a structured questionnaire. The midstream urine specimen was collected and cultured on cysteine-lactose-electrolyte-deficient agar and blood agar. Pure isolates were tested against the ten most prescribed antimicrobials using the Kirby-Bauer disk diffusion method. Data were entered and analysed using Statistical Program for Social Sciences version 21. A *p* value <0.05 was considered statistically significant.

**Results:**

The overall prevalence of significant bacteriuria was 23% (95% CI: 13.6, 26.8). The higher proportion of bacteria were isolated from pregnant women (14.1%) compared to nonpregnant women (8.9%). *Escherichia coli* (28.8%) and *Streptococcus aureus* (14.3%) were the most common isolates. *E. coli* was resistant to amoxicillin (83.3%), trimethoprim-sulfamethoxazole (78.6%), and ciprofloxacin (81%), whereas *S. aureus* was resistant to chloramphenicol (81%), erythromycin (81%), and amoxicillin (76.2%). Current symptoms, and history of catheterization increase the likelihood of urinary tract infections.

**Conclusion:**

Pregnant women were more likely infected with bacterial pathogens than nonpregnant women. Current symptoms, and catheterization increase the odds of urinary tract infections. More than half of the isolates were resistant to the commonly prescribed antimicrobials. Regular assessment of urinary tract infections and antimicrobial resistance are recommended to provide effective therapy and thereby prevent urinary tract complications.

## 1. Introduction

Urinary tract infection (UTI) refers to the presence and multiplication of bacterial pathogens in the organs of the urinary tract. It is characterized by a broad spectrum of symptoms ranging from mild irritative voiding to bacteremia, sepsis, or even death [1]. About 50 to 60% of women experience at least one episode of UTIs during their lifetime [2] because of the short length of the urethra along with proximity to the absence of bactericidal prostatic secretion and moist anal canal region [3].

Pregnant women are more likely infected with UTIs with uropathogenic bacteria than nonpregnant women and can be associated with adverse outcomes for both the mother and fetus. In pregnant women, the enlarged uterus affects all the tissues of the urinary tract at various degrees, while in the nonpregnant state, the uterus lies just behind and partly over the bladder. When the uterus grows, its weight increases and blocks the drainage of urine from the bladder, thus causing urinary stasis, which leads to infection of the urinary tract [4, 5]. Sexual intercourse also facilitates the ascent of bacteria into the bladder [6]. The UTIs are mainly caused by Gram-negative bacteria such as *Escherichia coli, Proteus* species, *Klebsiella* spp., and *Pseudomonas aeruginosa* and less extent by Gram-negative bacteria which include *Staphylococcus aureus, Enterococcus* spp., and *Streptococcus* spp. [7].

The emergence of antimicrobial resistance in the management of UTIs is a severe public health problem worldwide, particularly in developing countries where health care services are limited [4, 8]. The resistance is mainly due to the widespread use of antimicrobials in people and animal feeds. The resistance properties to antimicrobials are most often observed in hospital settings [9] and considered a challenge for clinicians due to the presence of limited treatment options [10].

In Ethiopia, UTI is common, as different researchers have reported [11–13]. Infection control and treatment measures mainly depend on the common etiology and their level of antimicrobial resistance in the local scenario [14]. Despite this, literature is scarce on the role of pregnancy in predisposition to urinary tract infections. This study was aimed to compare the magnitude of UTI, types of bacterial etiology, antimicrobial susceptibility patterns, and associated factors among pregnant and nonpregnant women.

## 2. Materials and Methods

### 2.1. Study Setting, Design, and Period

A health facility-based comparative cross-sectional study was conducted among pregnant and nonpregnant women attending public health facilities in Harar, Eastern Ethiopia, from February 2017 to December 2017. Harar is located at a distance of 515 km from Addis Ababa, the capital city of Ethiopia. According to the Harari Regional Health Bureau report of 2016, about 8400 pregnant and nonpregnant women received health care services, which are delivered through six hospitals, eight health centres, one Fistula centre, and more than 18 health posts (Harari Regional Health Bureau, 2016).

### 2.2. Participants and Sampling Procedure

Pregnant and nonpregnant women in the reproductive age group who come for health care services in the selected health facilities were qualified to participate in the study. Women treated with any type of antimicrobials 15 days before and during the study period were excluded from the study.

Double population proportion formula was employed to determine the sample size by taking the prevalence of UTIs among pregnant women (11%) and nonpregnant women (4.5%) from the study conducted in Nsukka, Nigeria [15], assuming a power of 80, and the ratio of exposed to unexposed 1 : 1. After 10% of a nonresponse rate was added, the final sample size was 651 (326 pregnant and 325 nonpregnant women).

**Figure 1:**
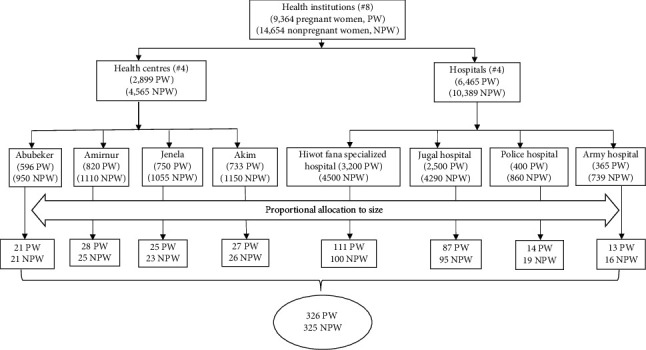
Sampling procedure for pregnant and nonpregnant women in public health facilities, Harar, Eastern Ethiopia, from February to December 2017.

The total sample size was distributed proportionally to the number of pregnant and nonpregnant women in those selected health facilities (Figure 1). A systematic random sampling technique was applied to select the study participants using the medical record as a sampling frame. After the 1^st^ participant was chosen by the lottery method, the subsequent was selected every K^th^ interval of individuals until the intended sample size has fulfilled.

### 2.3. Measurement and Data Collection Techniques

Sociodemographic characteristics (age, marital status, educational status, residence, occupation, income, etc.) and clinical data (the history of UTIs, current symptoms of UTIs, history of catheterization, and parity among others) were collected from eligible participants using a structured questionnaire. The questionnaire was translated into the local languages (Afan Oromo and Amharic). Three days of training was given for data collectors and supervisors on the purpose of the study, the quality of data, and the entire process of data management. The data were collected through face-to-face interviews by data collectors who fluently speak both languages. Other data, such as types of bacterial pathogens and their antimicrobial susceptibility profile, were collected from urine culture and antimicrobial susceptibility tests.

### 2.4. Sample Collection and Identification

The study participants were appropriately instructed about the required urine sample before collection. Five millilitres of midstream urine was collected from each participant using a wide-mouthed screw-capped universal sterile container. The specimen was immediately transported to the Microbiology Laboratory of the College of Health and Medical Sciences of Haramaya University for urine culture and antimicrobial susceptibility tests.

All urine samples were inoculated on cysteine-lactose-electrolyte-deficient agar and blood agar using a sterile calibrated wire loop (0.001 ml) (Oxoid Ltd., UK) and incubated at 37°C for 24 hours. The isolation of bacteria was made using colonial morphological features and Gram stain microscopy. Identification to the species level was made based on their biochemical reactions using analytical profile index (API) strips (BioMerieux® SA, MARCY I'Etolle, France). A 0.5 McFarland standard was prepared and dispensed into the API to rehydrate each well and incubated according to the manufacturer's instruction. All positive and negative test results are compiled to obtain a profile number, which is then compared with the profile numbers in the APIWEB™ database (or online) to determine the bacterial species. The colonies were counted and multiplied by 1000 to determine the number of bacteria per millilitre in the original urine specimen and checked for significant bacteriuria, which indicates the presence of UTIs [7].

### 2.5. Antimicrobial Susceptibility Testing

Antimicrobial susceptibility testing against ten antimicrobials was performed by the Kirby-Bauer disk diffusion method. Using a sterile wire loop, up to 5 pure colonies were picked and emulsified in normal saline (0.85% NaCl) until the suspension became equivalent to a 0.5 McFarland standard. A drop of the suspension was taken and evenly distributed over the entire surface of the Mueller Hinton agar (Oxoid Ltd., UK). The antimicrobial disks used to test against Gram-negative bacteria include amoxicillin (10 *μ*g), ceftazidime (30 *μ*g), cefoxitin (30 *μ*g), ceftriaxone (30 *μ*g), ciprofloxacin (5 *μ*g), gentamycin (10 *μ*g), and trimethoprim-sulfamethoxazole (1.25/23.75 *μ*g). Disks for Gram-positive bacteria contain the following antimicrobials: penicillin G (1 unit), amoxicillin (10 *μ*g), chloramphenicol (10 *μ*g), ceftazidime (30 *μ*g), cefoxitin (30 *μ*g), ceftriaxone (30 *μ*g), ciprofloxacin (5 *μ*g), erythromycin (15 *μ*g), and gentamycin (10 *μ*g). After incubation at 37°C for 18 to 24 hours, the zone diameter of inhibition was measured to the nearest millilitre and interpreted as sensitive (S), intermediate (I), or resistance (R) per the Clinical and Laboratory Standards Institute (CLSI) guideline [16]. All antimicrobials obtained from Oxoid Ltd. Bashingstore Hampshire, UK.

### 2.6. Data Quality Control

Before the actual work, training was given for data collectors on the data collection procedure and sample examination by the investigators. The structured questionnaire first prepared in the English language was translated into Amharic and Afan Oromo by the bilingual expert and back-translated by another bilingual expert. This tool was pretested prior to data collection at Dilchora Hospital and Sabian Health Centre, Dire Dawa, Ethiopia. During pretesting, additional information was gathered and recognized vague terms, phrases, and questions were modified. Culture media were prepared following the respective manufacturer's instructions. Media were checked for sterility and viability using *E. coli* (ATCC 25922), *S. aureus* (ATCC 25923), and *P. aeruginosa* (ATCC 27853) reference strains. The inoculums density of the bacterial suspension was standardized to 0.5 McFarland standard. During the whole phase of the study period, the research activities were supervised by the investigators and, thereby, any doubt cleared on the spot. The collected data were reviewed and checked every day for completeness.

### 2.7. Data Processing and Analysis

Data were entered into EpiData version 3.1 and exported to the Statistical Package for Social Sciences version 21 (SPSS, Inc., Chicago, Ill., USA) software for analysis. The analysis included both descriptive and inferential statistics. The descriptive statistics such as percentage, mean, and standard deviation were calculated to describe the independent variables. Variables with *p* < 0.2 on a bivariate logistic regression analysis were candidates for a multivariate logistic regression analysis model, which accounts for potential confounding and identification of independent predictors of UTIs. The data were summarized by employing the odds ratio and 95% confidence interval. Finally, a predictor variable with a *p* value <0.05 was considered a statistically significant association.

### 2.8. Ethical Consideration

Ethical clearance was obtained from the Institutional Health Research Ethics Review Committee of the College of Health and Medical Sciences, Haramaya University. Permission to conduct the study was also secured from selected health facilities. Important information about the purpose of the study, its potential contribution, and procedures was explained to the participants with the assurance of maintaining their confidentiality. The participants take part in the study based on their willingness and ability to give informed consent, and they also assured that disagreements and discontinuations from the study had no effect on the services to be provided. The interviews were done after getting written informed consent from each participant.

## 3. Results

### 3.1. Sociodemographic Characteristics

A total of 638 women (319 pregnant and 319 nonpregnant) were participated in this study, with a response rate of 98%. The age of participants ranged from 18 to 49 years, with a mean of 33 (±5.6 standard deviation). The majority of the participants (59.7%) were in the age group of 21–30 years. The educational status of the participants varied from no formal education to postgraduate studies. Nearly half (45.6%) of the participants attended primary school (18^th^ grade). The majority of the participants were married (60.8%), rural dwellers (71.8%), merchants (40.3%), and earn a monthly income of less than 59.00 US dollars (USD) (45.9%) (Table 1).

### 3.2. Distribution and Bacterial Etiologies

Of the 638 urine specimens, 147 were found to be positive for culture, and the overall prevalence of UTIs was 23% (95% CI: 13.6, 26.8). Higher significant bacteriuria was detected in pregnant women (14.1%) than nonpregnant women (8.9%), with a statistically significant difference (*χ*2 = 0.63, *d* = 1, *p* = 0.003). Of the total isolates, Gram-negative bacteria were more prevalent, 100 (68%), than Gram-positive bacteria, 47 (32%). The predominantly isolated bacteria were *E. coli* (28.8%) followed by *S. aureus* (14.3%) (Table 2).

### 3.3. Antimicrobial Susceptibility Testing

Gram-negative bacterial isolates (*n* = 100) showed a resistance rate of 70% to trimethoprim-sulfamethoxazole, 61% to ciprofloxacin, and 60% to amoxicillin. *Escherichia coli* were 83.3% resistant to amoxicillin, 81% to ciprofloxacin, 78.6% to trimethoprim-sulfamethoxazole, and 57.1% to ceftriaxone, whereas 92.9% were sensitive to ceftazidime, 83.3% to cefoxitin, and 81% to gentamicin. *Proteus* spp. were 87.5% resistant to ciprofloxacin and 56.2% to trimethoprim-sulfamethoxazole, while 100% were sensitive to ceftazidime, 93.8% to cefoxitin, and 87.5% to ceftriaxone. *Klebsiella* spp. were 86.7% resistant to amoxicillin and 73.3% to trimethoprim-sulfamethoxazole but 93.3% were sensitive to ciprofloxacin, 73.3% to ceftazidime, and 66.7% to ceftriaxone (Table 3).

Gram-positive bacteria (*n* = 47) were resistant to amoxicillin (72.3%), chloramphenicol (68.1%), and erythromycin (59.5%). Of these, the most common isolates *S. aureus* (44.7%) showed resistance to most antimicrobial agents. The level of resistance of *S. aureus* to both chloramphenicol and erythromycin was high (81%) followed by amoxicillin (76.2%) but was sensitive to penicillin G (85.7%), cefoxitin (85.7%), ciprofloxacin (85.7%), and gentamicin (71.4%). *S. saprophyticus* were resistant to amoxicillin (75%), ceftazidime (68.8%), and gentamicin (68.8%) (Table 4).

### 3.4. Factors Associated with Urinary Tract Infections

In the bivariate logistic regression analysis, age, residence, educational status, occupation, income, history of UTI, current symptoms of UTI, and history of the catheterization and/or indwelling were significantly associated with UTIs. In multivariate analysis, current symptoms of UTIs and history of catheterization and/or indwelling were significantly associated with UTIs. Women with current symptoms of UTIs were 11 times more likely to have UTIs (AOR: 11.2, 95% CI: 6.3, 19.8) than asymptomatic women. Women who had the history of catheterization and/or indwelling were 18 times more likely infected by UTIs compared to noncatheterized women (AOR: 18.4, 95% CI: 8.7, 38.5). However, there was no statistically significant association between UTIs and maternal age, residence, educational status, occupation, parity, history of hospitalization, and genital washing with soap after adjustment for confounding factors (Table 5).

## 4. Discussion

In the present study, the overall prevalence of UTIs among both pregnant and nonpregnant women was 23% (95% CI: 13.6, 26.8). The prevalence of UTIs among pregnant and nonpregnant was 14.1% and 8.9%, respectively. Current symptoms of UTIs, and the history of catheterization and/or indwelling were the factors significantly associated with UTIs.

The findings indicated that the overall prevalence of UTIs among women was higher, and it was more prevalent in pregnant women than nonpregnant women, indicating that UTIs are rampant during pregnancy and highlight the burden of the problem, which necessitates appropriate intervention. This result was comparable with the studies reported from India (17.5% pregnant versus 5.7% nonpregnant women) [17], Kenya (14.2%) [18], and Nigeria (11% pregnant versus 4.5% nonpregnant) [15]. However, the prevalence reported in the current study was low compared with the result from Nigeria (40% pregnant versus. 31% nonpregnant) [19]. Perhaps the susceptibility to UTIs during pregnancy is due to urethral dilation [20]; the environment, low socioeconomic status, poor personal hygiene, and hormonal & physiological changes could also contribute to the risk of UTIs [19].

In this study, the history of catheterization and/or indwelling was shown to increase the odds of UTIs in women. The finding was supported by different studies [5, 13]. The higher prevalence of UTIs detected among asymptomatic women, on the other hand, is of significant concern since most of the women (67%) did not have complaints suggestive of UTIs. Due to this, clinicians may miss these cases and complications may ensue later in an infected individual. The best ways of preventing catheter-associated urinary tract infections are to avoid unnecessary use of a catheter, maintaining strict techniques during insertion, and their prompt removal when they are no longer needed [19, 20].

Gram-negative bacteria were more prevalent than Gram-positive (68%) in the present study. Similar findings were reported in studies conducted elsewhere: Erode, India [21], Owerri, Nigeria [19], and Sagamu, Nigeria [22]. This was inconsistent with a study conducted at Hawassa, Ethiopia, which reported a higher prevalence of Gram-positive bacteria (51%) [12]. The preponderance of Gram-negative bacteria might be due to the presence of adhesions that help for the attachment to the uroepithelial cells and prevent bacteria from urinary lavage, allowing for multiplication and tissue invasion resulting in pyelonephritis in pregnancy [19, 23].

The most predominant causative agent of UTIs in this study was *E. coli* (28.8%) followed by *S. aureus* (14.3%), which was similar in etiology but different in magnitude with previous findings reported in Ethiopia as well as in other parts of the world such as in Benin, Nigeria (18.52%) [24], Gondar, Ethiopia (47.5%) [11], and Mulago, Uganda (57.5%) [25]. It disagreed with the study conducted at Hawassa, Ethiopia, which showed higher coagulase-negative staphylococci (32.6%) than *E. coli* (26.1%) [12]. The higher rate of *E. coli* isolation might be attributed to the varieties of virulence factors such as P-fimbria and S-fimbria adhesions for colonization and invasion of the urinary epithelium and persistence in the vagina [19, 23].

*Escherichia coli* showed 83.3% resistance to amoxicillin, 78.6% to trimethoprim-sulfamethoxazole, and 81% to ciprofloxacin, and this implies that these drugs are not recommended to use as empirical therapy for UTI, particularly in this study area. However, ceftazidime, cefoxitin, and gentamicin could be considered for the treatment of UTIs. Resistance among bacterial uropathogens to commonly used antimicrobials becomes increasing, which makes clinicians with very few choices of drugs for the treatment of UTIs [4, 8]. On the other hand, *S. aureus* was the most dominant resistant organism to a number of antimicrobials like chloramphenicol (81%), erythromycin (81%), and amoxicillin (76.2%) but was sensitive to penicillin G (85.7%), cefoxitin (85.7%), ciprofloxacin (85.7), and gentamicin (71.4%). The development of resistance in bacteria might be due to the frequent use of broad-spectrum antimicrobials in people and animal feeds [9], inappropriate and incorrect administration of antimicrobial agents in empiric therapies, and lack of appropriate infection control strategies, which can cause a shift of resistant organisms in the hospital and the community.

This study was based on health facility and may not reflect the broader situation in the community. Anaerobic bacteria, fungi, and viral agents causing UTIs were not investigated due to limited laboratory facilities. The antimicrobial sensitivity test against bacteria is an *in-vitro* activity and may not reflect an *in-vivo* activity. Because susceptibility rates vary among health institutions, the findings may not be representative and reproducible in other health institutions. There may also be some observational errors, especially measuring the antimicrobial inhibition zone. Despite these limitations, the study provides ample up-to-date evidence regarding UTIs in pregnant and nonpregnant women, antimicrobial susceptibility profiles, and their associated factors.

## 5. Conclusions

The overall prevalence of UTIs among women was higher, and pregnant women had a higher risk of UTIs than nonpregnant women. Current symptoms of UTIs, and history of catheterization were predictors of UTIs. The most common causative agents of UTIs were *E. coli* and *S. aureus*. Most of the isolates were resistant to commonly prescribed antimicrobial agents. Regular screening of women for UTIs, particularly pregnant women, is recommended to discover the infected cases and on-time treatment to avoid urinary complications. There is a need to develop locally relevant guidelines for the management of UTIs considering issues like susceptibility patterns of bacteria causing UTIs in the local population and to supervise antimicrobial usage to promote rational drug use. Besides, further exploration is recommended to cover unaddressed issues by the present study.

## Figures and Tables

**Table 1:** Sociodemographic characteristics of pregnant and nonpregnant women at public health facilities, Harar, Eastern Ethiopia, from February 2017 to December 2017.

Variables	Pregnant, *n* (%)	Nonpregnant, *n* (%)	Total, *n* (%)
Age (years)			
>40	4 (36.4)	7 (63.6)	11 (1.7)
31–40	94 (46.1)	110 (53.9)	204 (32)
21–30	205 (53.8)	176 (46.2)	381 (59.7)
<21	16 (38.1)	26 (61.9)	42 (6.6)
Educational status			
No formal education	53 (47.3)	59 (52.7)	112 (17.6)
Primary school (1-8^th^)	140 (48.1)	151 (51.9)	291 (45.6)
Secondary school (9–12^th^)	55 (49.5)	56 (50.5)	111 (17.4)
Above secondary school	71 (57.3)	53 (42.7)	124 (19.4)
Marital status			
Married	200 (51.5)	188 (48.5)	388 (60.8)
Divorced	68 (46.6)	78 (53.4)	146 (22.9)
Widowed	38 (55.1)	31 (44.9)	69 (10.8)
Single	13 (37.1)	22 (62.9)	35 (5.5)
Residence			
Urban	94 (52.2)	86 (47.8)	180 (28.2)
Rural	225 (49.1)	233 (50.9)	458 (71.8)
Occupation			
Employee	16 (41)	23 (59)	39 (6.1)
House-wife	103 (53.1)	91 (46.9)	294 (30.4)
Daily labor	83 (56.1)	65 (43.9)	148 (23.2)
Merchants	117 (45.5)	140 (54.5)	157 (40.3)
Average monthly income (USD)			
>117.00	75 (52.1)	69 (47.9)	144 (22.1)
59.00–117.00	115 (57.2)	86 (42.8)	201 (31.5)
<59.00	129 (44)	164 (56)	293 (45.9)

**Table 2:** Types of bacteria species isolated from the urine of pregnant and nonpregnant women at public health facilities, Harar, Eastern Ethiopia, from February 2017 to December 2017.

Bacteria isolates	Pregnant (*n* = 90), number of positive (%)	Nonpregnant (*n* = 57), number of positive (%)	Total (*n* = 147), *n* (%)
*E. coli*	24 (26.7)	18 (31.8)	42 (28.8)
*S. aureus*	13 (14.4)	8 (14)	21 (14.3)
*Enterobacter* spp.	8 (8.9)	3 (5.3)	11 (7.4)
*Klebsiella* spp.	9 (10)	6 (10.5)	15 (10.2)
*S. saprophyticus*	12 (13.3)	4 (7)	16 (10.8)
*P. aeruginosa*	7 (7.8)	4 (7)	11 (7.4)
*Proteus* spp.	10 (11.1)	6 (10.5)	16 (10.8)
CoNS	3 (3.3)	1 (1.8)	4 (2.7)
Group B streptococcus	2 (2.2)	4 (7)	6 (4.1)
*Acinetobacter* spp.	2 (2.2)	3 (5.2)	5 (3.4)

CoNS: Coagulase-negative staphylococcus.

**Table 3:** Antimicrobial susceptibility pattern of Gram-negative bacteria isolated from the urine of pregnant and nonpregnant women at public health facilities, Harar, Eastern Ethiopia, from February 2017 to December 2017.

Bacteria isolates	*n* (%)	Pattern	Antimicrobial susceptibility testing, *n* (%)						
			AML	CAZ	FOX	CRO	CIP	CN	STX
*E. coli*	42 (42)	S	7 (16.7)	39 (92.9)	35 (83.3)	18 (42.9)	7 (16.7)	34 (81)	8 (19)
		I	0 (0)	0 (0)	4 (9.5)	0 (0)	1 (2.4)	1 (2.4)	1 (2.4)
		R	35 (83.3)	3 (7.1)	3 (7.1)	24 (57.1)	34 (81)	7 (16.7)	33 (78.6)
*Enterobacter* spp.	11 (11)	S	3 (27.3)	10 (90.9)	6 (54.5)	11 (100)	1 (9.1)	10 (90.9)	7 (63.6)
		I	0 (0)	0 (0)	4 (36.4)	0 (0)	0 (0)	0 (0)	0 (0)
		R	8 (72.7)	1 (9.1)	1 (9.1)	0 (0)	10 (90.9)	1 (9.1)	4 (36.4)
*Acinetobacter* spp.	5 (5)	S	2 (40)	5 (100)	5 (100)	1 (20)	4 (80)	5 (100)	1 (20)
		I	1 (20)	0 (0)	0 (0)	0 (0)	0 (0)	0 (0)	0 (0)
		R	2 (40)	0 (0)	0 (0)	4 (80)	1 (20)	0 (0)	4 (80)
*Klebsiella* spp.	15 (15)	S	2 (13.3)	11 (73.3)	9 (60)	10 (66.7)	14 (93.3)	8 (53.3)	3 (20)
		I	0 (0)	0 (0)	1 (6.7)	0 (0)	1 (6.7)	1 (6.7)	1 (6.7)
		R	13 (86.7)	4 (26.7)	5 (33.3)	5 (32.3)	0 (0)	6 (40)	11 (73.3)
*P. aeruginosa*	11 (11)	S	2 (18.2)	10 (90.9)	9 (81.8)	5 (45)	9 (81.8)	4 (36.4)	1 (9.1)
		I	0 (0)	1 (9.1)	1 (9.1)	0 (0)	0 (0)	0 (0)	0 (0)
		R	9 (81.8)	0 (0)	1 (9.1)	6 (54.5)	2 (18.2)	7 (63.6)	11 (90.9)
*Proteus* spp.	16 (16)	S	13 (81.3)	16 (100)	15 (93.8)	14 (87.5)	1 (6.2)	5 (31.2)	7 (43.3)
		I	0 (0)	0 (0)	1 (6.3)	0 (0)	1 (6.2)	6 (37.6)	0 (0)
		R	3 (18.8)	0 (0)	0 (0)	2 (12.5)	14 (87.5)	5 (31.2)	9 (56.2)
Total, *n* (%)	100	S	29 (29)	91 (91)	79 (79)	59 (59)	36 (36)	66 (66)	27 (27)
		I	1 (1)	1 (1)	11 (11)	0 (0)	3 (3)	8 (8)	3 (3)
		R	70 (60)	8 (8)	10 (10)	41 (41)	61 (61)	26 (26)	70 (70)

S: sensitive; I: intermediate; R: resistance; AML: amoxicillin; CAZ: ceftazidime; FOX: cefoxitin; CIP: ciprofloxacin; CN: gentamicin; CRO: ceftriaxone; STX: trimethoprim-sulfamethoxazole.

**Table 4:** Antimicrobial susceptibility pattern of Gram-positive bacteria isolated from the urine of pregnant and nonpregnant women at public health facilities, Harar, Eastern Ethiopia, from February 2017 to December 2017.

Bacteria isolates	*n* (%)	Pattern	Antimicrobial susceptibility testing, *n* (%)								
			*P*	AML	C	CAZ	FOX	CRO	CIP	E	CN
*S. aureus*	21 (44.7)	S	18 (85.7)	5 (23.8)	4 (19)	14 (66.7)	18 (85.7)	16 (76.2)	17 (85.7)	4 (19)	15 (71.4)
		I	0 (0)	0 (0)	0 (0)	0 (0)	0 (0)	0 (0)	1 (4.8)	0 (0)	0 (0)
		R	3 (14.3)	16 (76.2)	17 (81)	7 (33.3)	3 (14.3)	5 (23.8)	2 (9.5)	17 (81)	6 (28.6)
*S. saprophyticus*	16 (34)	S	9 (56.3)	4 (25)	9 (56.2)	5 (31.3)	10 (62.5)	10 (62.5)	14 (87.5)	10 (62.5)	5 (31.2)
		I	3 (18.8)	0 (0)	0 (0)	0 (0)	4 (25)	3 (18.8)	1 (6.3)	1 (6.2)	0 (0)
		R	4 (25)	12 (75)	7 (43.8)	11 (68.8)	2 (12.5)	3 (18.8)	1 (6.3)	5 (31.2)	11 (68.8)
CoNS	4 (8.5)	S	3 (75)	2 (50)	1 (25)	3 (75)	4 (100)	3 (75)	2 (50)	1 (25)	3 (75)
		I	1 (25)	0 (0)	0 (0)	0 (0)	0 (0)	0 (0)	0 (0)	0 (0)	0 (0)
		R	0 (0)	2(50)	3 (75)	1 (25)	0 (0)	1 (25)	2 (50)	3 (75)	1 (25)
Group B streptococcus	6 (12.8)	S	5 (83.3)	1 (16.7)	1 (16.7)	3 (50)	3 (50)	3 (50)	3 (50)	2 (33.3)	3 (50)
		I	0 (0)	0 (0)	0 (0)	1 (6.7)	2 (33.3)	0 (0)	1 (6.7)	1 (16.7)	0 (0)
		R	1 (16.7)	5 (83.3)	5 (83.3)	2 (33.3)	1 (6.7)	3 (50)	2 (33.3)	3 (50)	3 (50)
Total, *n* (%)	47 (100)	S	35 (74.5)	13 (27.7)	15 (31.9)	24 (51.1)	35 (74.5)	32 (68.1)	31 (66)	17 (36.2)	26 (55.3)
		I	4 (8.5)	0 (0)	0 (0)	1 (2.1)	2 (4.3)	5 (10.6)	3 (6.4)	2 (4.3)	0 (0)
		R	8 (17)	34 (72.3)	32 (68.1)	22 (46.8)	10 (21.3)	10 (21.3)	13 (27.7)	28 (59.5)	21 (44.7)

S: sensitive; I: intermediate; R: resistance; P: penicillin G; AML: amoxicillin; C: chloramphenicol; CAZ: ceftazidime; FOX: cefoxitin; CRO: ceftriaxone; CIP: ciprofloxacin; E: erythromycin; CN: gentamicin.

**Table 5:** Factors associated with UTIs among pregnant and nonpregnant women at public health facilities, Harar, Eastern Ethiopia, from February 2017 to December 2017.

Variables	Significant bacteriuria		Crude OR (95% CI)	Adjusted OR (95% CI)
	Yes (%)	No (%)		
Age group (in years)				
>40	6 (54.5)	5 (45.5)	0.19 (0.1, 0.8)	0.28 (0.1, 1.6)
31–40	49 (24)	155 (76)	0.74 (0.3, 1.7)	1.26 (0.5, 3.3)
21–30	84 (22)	297 (78)	0.83 (0.4, 1.9)	1.11 (0.5, 2.8)
<21	8 (19)	34 (81)	1	1
Residence				
Rural	34 (18.9)	146 (81.1)	0.71 (0.9, 2.2)	1.14 (0.7, 1.9)
Urban	113 (24.7)	345 (75.3)	1	1
Educational status				
No formal education	19 (17)	93 (83)	0.98 (0.5, 2.0)	1.52 (0.7, 3.3)
Primary school	66 (22.7)	22 5(77.3)	1.37 (0.7, 2.7)	1.01 (0.5, 1.9)
Secondary school	30 (27)	81 (73)	1.54 (0.7, 3.2)	0.85 (0.4, 1.8)
Above secondary school	32 (25.8)	92 (74.2)	1	1
Occupation				
Employee	16 (71)	23 (59)	0.41 (0.2, 0.8)	0.71 (0.2, 2.7)
House-wife	48 (24.7)	146 (75.3)	0.87 (0.6, 1.4)	1.15 (0.4, 2.9)
Daily labour	26 (17.6)	122 (82.4)	1.34 (0.8, 2.2)	1.44 (0.7, 3.2)
Merchants	57 (22.2)	200 (77.8)	1	1
Monthly income				
>117.00 USD	45 (32.4)	94 (67.6)	0.56 (0.4, 0.9)	0.51 (0.3, 1.9)
59.01–117.00 USD	38 (19.4)	158 (80.6)	1.10 (0.7, 1.7)	0.88 (0.5, 1.5)
<59.00 USD	64 (21.1)	239 (78.9)	1	1
Parity				
Nullipara	38 (23.8)	122 (76.3)	1	
Multipara	109 (22.8)	369 (77.2)	1.05 (0.7, 1.6)	
History of hospitalization				
Yes	17 (28.3)	43 (71.7)	0.73 (0.4, 1.3)	
No	130 (22.5)	448 (77.5)	1	
History of UTI				
Yes	28 (27.5)	74 (72.5)	1.33 (0.5, 1.2)	1.06 (0.6, 1.9)
No	119 (22.2)	417 (77.8)	1	1
Current symptoms of the UTI				
No	65 (67)	32 (33)	1	1
Yes	82 (15.2)	459 (84.8)	11.37 (7.0, 18.5)	11.20 (6.3, 19.8)^∗^
History of catheterization				
No	48 (80)	12 (20)	1	1
Yes	99 (17.1)	479 (82.9)	19.35 (9.9, 37.8)	18.36 (8.7, 38.5)^∗^
Genital wash with soap				
Yes	130 (25.7)	375 (74.3)	1	
No	17 (12.8)	116 (82.2)	2.37 (1.4, 4.1)	

*Note.* ^*∗*^Statistically significant; OR: odds ratio; CI: confidence interval.

## Data Availability

The SPSS data used to support the findings of this study are included within the article.
